# Unveiling the nexus between perceived overqualification and knowledge hiding: Moderated mediation analysis of job crafting and job boredom

**DOI:** 10.1016/j.heliyon.2024.e31701

**Published:** 2024-05-22

**Authors:** Jawad Khan, Qingyu Zhang, Imran Saeed, Amna Ali, Mohammad Fayaz

**Affiliations:** a Research Institute of Business Analytics and Supply Chain Management, College of Management, Shenzhen University, Shenzhen, China; b Institute of Business and Management Sciences, The University of Agriculture, Peshawar, Pakistan; c Department of Business Administration, IQRA National University, Peshawar, Pakistan

**Keywords:** Perceived overqualification, Job crafting, Knowledge hiding, Job boredom, Relative deprivation theory

## Abstract

**Purpose:**

Grounding on relative deprivation theory, this study aimed to investigate the relationship between perceived overqualification (POQ) and knowledge hiding. Furthermore, this study investigated the mediating role of job boredom and the moderating effect of job crafting.

**Design:**

/Methodology/Approach: This study employs Hayes’ PROCESS model to analyze data obtained from 374 employees working in the hospitality and tourism industry.

**Findings:**

The results indicate a positive relationship between POQ, job boredom, and knowledge hiding. These findings suggest that job boredom mediates the relationship between POQ and knowledge hiding. Furthermore, the study showed a moderated mediation path wherein the interaction effect of POQ and job crafting on knowledge hiding was mediated by job boredom.

**Research limitations/implications:**

Data were collected from the hospitality and tourism industry, limiting the generalizability of the findings to other sectors. Additionally, the study relied on self-reported measures, which may have resulted in a bias.

**Practical implications:**

Conduct thorough job fit assessments during the hiring process to ensure that candidates' qualifications align closely with job requirements. By matching employees' skills and experiences to their job, organizations can reduce perceived overqualification, which may lower job boredom and knowledge hiding tendencies.

**Originality/value:**

This study's focus on person-job misfits adds a new layer of insight into employee experiences in the workplace. By examining how mismatches between individuals and their roles contribute to job boredom and knowledge hiding, this study highlights the importance of aligning job responsibilities with employee skills, qualifications, and preferences.

## 1 Introduction

Knowledge plays a paramount role in the hospitality and tourism industry, and influences its success and sustainability. A deep understanding of customer preferences, cultural nuances, and emerging trends is essential to provide exceptional guest experiences. Knowledge also aids in effective resource management, optimizing operational efficiency, and maintaining high service standards [1]. Recognizing that employees are the primary source of knowledge management, many businesses in this sector seek to acquire exceptional talent with greater degrees of knowledge to achieve sustainable growth in the market [2]. Hospitality and tourism organizations understand that the knowledge and expertise possessed by their employees directly contribute to quality of service, guest satisfaction, and overall competitiveness [3].

Consequently, hospitality and tourism organizations strive to attract and retain employees with specialized knowledge, valuable skills, and a deep understanding of the industry [4]. In the hospitality and tourism industry, there is a growing trend to increase the recruitment threshold to attract individuals with better educational backgrounds and higher knowledge levels [5]. Organizations in service sector recognize the value of employees with advanced education and knowledge in driving innovation, enhancing service quality, and achieving sustainable growth [6,7]. Setting high educational requirements for certain positions may unintentionally lead to overqualification “a condition in which an individual's believe they have an excess of knowledge, skills, and abilities (KSAs) compared to what is necessary for their positions” [8].

However, it is important to consider that overqualified employees may not always be willing to share knowledge with their employees [9]. Erdogan, Karakitapoğlu‐Aygün [10] found a negative association between perceived overqualification (POQ) and helping behaviors towards employees. Furthermore, there is a possibility that overqualified employees may intentionally hide their knowledge from their peers [11]. Although recent studies have indicated a potential relationship between POQ and knowledge hiding [12,13], empirical investigations have yet to explore the nature of this association. Consequently, this study examines the connection between POQ and knowledge hiding, which refers to deliberately withholding requested knowledge from others [11]. Building upon the relative deprivation theory [14], we argue that overqualified employees who may experience a sense of relative deprivation [15], may choose to express their frustration by hiding knowledge [16].

Furthermore, by expanding our study model, we introduce an underlying mechanism, “job boredom.” When employees find themselves overqualified, where they possess more skills and abilities than required for their current job, it can lead to a phenomenon known as “job boredom.” Job boredom refers to the subjective experience of feeling unengaged, unchallenged, and dissatisfied with one's work tasks [17]. Boredom is an aversive emotional state characterized by a lack of interest in, or stimulation of, one's surroundings. In the context of overqualification, employees may feel unfulfilled and unstimulated because their skills and abilities are not fully utilized or challenged in their current roles. This sense of underutilization and monotony can trigger negative emotions, such as frustration, restlessness, and dissatisfaction [18]. Employees who experience persistent boredom may engage in knowledge hiding as a coping mechanism to counteract the negative effects of boredom. When individuals feel unengaged or unchallenged at work, they may perceive their knowledge as a source of power or control. By intentionally withholding their knowledge from others, they may exert control over their work environment and create a perceived advantage for themselves [19]. According to relative deprivation theory, individuals who perceive a mismatch between their qualifications and job requirements may experience boredom. The theory suggests that a lack of alignment between an individual's skills, abilities, and qualifications and the demands and challenges of their job can lead to feelings of deprivation and dissatisfaction [20].

In addition, we introduced a buffering mechanism called “job crafting” to mitigate this link. We argue that in the workplace, overqualified individuals often encounter situations in which there might be a misalignment between their skills, qualifications, personal motivations, and the requirements or nature of their job. Recognizing this misalignment, employees may choose to employ a buffering mechanism known as “job crafting” to actively shape and redefine their work experiences [21,22]. Job crafting is a deliberate and proactive approach wherein employees take control of their work environment to enhance various aspects of their professional lives [23,24]. The fundamental idea behind job crafting is to create a better alignment between an individual's skills, interests, and job requirements [25]. This process is not about seeking a new position but rather about modifying existing tasks, relationships, and perceptions associated with the current role [26,27].

**Fig. 1:**
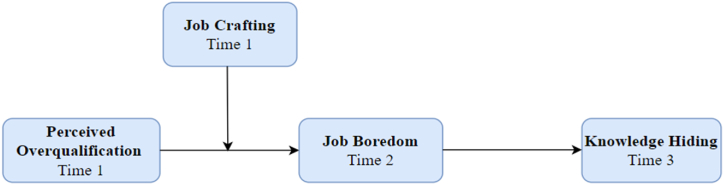
Theoretical framework.

The research model is illustrated in Fig. 1 contributes to the literature in many ways. First, while previous research has examined the negative consequences of POQ, such as job dissatisfaction and turnover intention, this study extends this understanding by investigating the relationship between POQ and knowledge hiding in the hospitality and tourism sectors. By focusing on intentional knowledge withholding, this study sheds light on how overqualified employees may hide their knowledge in response to perceived relative deprivation. Second, this study introduced job boredom as a mediating mechanism between POQ and knowledge hiding. This suggests that overqualified employees who experience a person-job misfit are more prone to engage in knowledge-hiding behaviors. By considering job boredom as a mediating factor, this study provides insights into the underlying psychological processes that drive knowledge-hiding among overqualified employees. Third, by introducing job crafting as a buffering mechanism, this study offers a potential solution to mitigate the negative consequences of POQ, such as knowledge hiding. This suggests that when employees have the opportunity to craft their jobs and make changes that better utilize their surplus knowledge and skills, they are more likely to experience a sense of engagement and fulfillment. In turn, this may reduce their inclination to engage in knowledge-hiding behaviors.

## 2 Literature review and hypotheses development

### 2.1 POQ and job boredom

Employees with surplus qualifications and skills often seek roles that provide them with opportunities to utilize and develop these skills further. They may have higher expectations for their job roles because they have invested time and effort in acquiring those qualifications, and they naturally want roles that can fully utilize their abilities [28]. When overqualified employees are not intellectually or creatively challenged in their roles, it can lead to boredom and disengagement. This lack of stimulation can result in decreased motivation and job satisfaction over time [29]. The misfit between their qualifications and the assigned tasks can erode their motivation. They might start to feel that their skills are wasted, which can lead to a negative attitude towards their job [30].

According to relative deprivation theory, when employees feel overqualified, they may find that their job tasks are not intellectually stimulating or challenging enough to fully engage their skills and knowledge. As a result, they may become bored and uninterested in their work [31]. POQ can create a sense of stagnation and limited growth prospects. If employees believe that their current jobs do not provide opportunities for advancement or skill development, they may become bored and disengaged over time [32]. Overqualified individuals may find themselves performing tasks monotonously, routinely, or below their skill levels. Engaging in repetitive work without the opportunity to utilize their full potential can lead to feelings of boredom and disengagement [33]. Employees with an overqualification set that is not fully utilized in their current roles may feel frustrated or unfulfilled. The mismatch between their capabilities and their job demands can contribute to boredom and dissatisfaction [19,34]. POQ can negatively impact an individual's motivation. Overqualified employees feel that their skills are not being utilized or appreciated. They may experience a decline in motivation to perform at their best, leading to a lack of interest in their jobs and increased boredom [32]. Overqualified individual may also perceive limited potential for career advancement or growth in their current position. This perception can further contribute to boredom, as they may feel stuck or unfulfilled in their jobs without a clear path for progression [35].

Hypothesis 1 There is a positive relationship between POQ and job boredom

### 2.2 POQ and knowledge hiding

POQ refers to individuals' perception that their qualifications, skills, or experiences exceed the requirements of their current job roles. This often leads to feelings of underutilization, frustration, and dissatisfaction among employees [36]. Relative deprivation theory posits that POQ often triggers social comparison in the workplace. Individuals compare their qualifications, skills, and experiences with those of their colleagues and peers. If they perceive themselves as more qualified or competent than their peers but are not appropriately recognized or utilized within the organization, feelings of relative deprivation may arise. This sense of inequity can fuel the desire to retaliate or protect oneself, leading to knowledge hiding as a way to regain a sense of control or power within the organization [33]. POQ can also threaten an individual's status and identity within the organization. When overqualified individuals feel that their qualifications are not adequately recognized or valued by their superiors or colleagues, they can undermine their sense of self-worth and identity as competent professionals [20]. In response, overqualified individuals may engage in knowledge hiding as a means of self-protection. By withholding their expertise or information, they may attempt to maintain a sense of superiority or control over their domain of knowledge [11].

Overqualified individuals may fear that if they openly share their knowledge and capabilities, this could render them less essential or even expendable in the organization. They may worry that others will catch up with their level of competence, thus making their unique qualifications less valuable. Consequently, they may hide their knowledge to protect their positions [37]. Overqualified feelings can lead to lack of motivation or engagement. When individuals believe that their skills and abilities are underutilized, they may develop a sense of apathy or detachment. Consequently, they may be less inclined to actively contribute their knowledge and ideas to the organization, resulting in knowledge hiding [8]. According to relative deprivation theory, overqualified individuals may feel deprived because they believe they deserve better or more challenging opportunities that align with their qualifications. This perception of relative deprivation can lead to negative emotions, such as frustration, dissatisfaction, or resentment. Consequently, individuals may hide knowledge to protect their perceived advantage or gain a sense of control over their work environment [38].

Hypothesis 2 POQ is positively related to knowledge hiding

### 2.3 Job boredom as a mediator

POQ can negatively affect individuals' motivation to perform well in their jobs. When individuals feel that their skills and abilities are not fully utilized or recognized, they may lose motivation to exert effort and engage in their work, resulting in boredom [39]. According to person-job fit theory, misalignment between an individual's qualifications and job responsibilities may lead to job boredom [11]. Overqualified individuals for their job roles may find their daily tasks too easy or routine, lacking the level of challenge they desire. Without challenging and engaging in work, individuals may quickly become bored and disengaged from their job responsibilities [37]. When individuals believe that they are overqualified for their job roles, they often experience a sense of underutilization, feeling that their skills, knowledge, and abilities are not being fully utilized or recognized. This underutilization can lead to feelings of frustration and dissatisfaction with their work. This, in turn, can fuel knowledge hiding behaviors, as individuals perceive that their efforts to share knowledge are not valued or appreciated within the organization [40].

As overqualified individuals become disengaged and disenchanted with their work, they may feel less motivated to contribute their knowledge and expertise to the organization. They may perceive that their efforts to share knowledge are not valued or appreciated, particularly if they feel that their qualifications exceed the demands of their job. Consequently, they may withhold valuable information or expertise from their colleagues or the organization as a form of passive resistance or self-protection [41]. According to Arar [42], when employees feel undervalued or unappreciated in their roles, they may be less inclined to share their knowledge with others, fearing that their contributions will go unrecognized or unacknowledged. This reluctance to share knowledge can impede collaboration, hinder organizational learning, and stifle innovation, ultimately undermining an organization's ability to adapt and thrive in a dynamic and competitive environment [43].

Hypothesis 3 Job boredom serves as a mediating factor between POQ and knowledge hiding

### 2.4 Job crafting as a moderator

When employees perceive themselves as overqualified for their current jobs, they often experience monotony and boredom because of underutilization of their skills and capabilities. However, through job crafting, individuals can take proactive steps to seek new and challenging tasks that align with their skills and interests, thereby combating the negative effects of overqualification [44]. By taking on additional responsibilities, employees can expand their job scope and engage in tasks that utilize their expertise. They can identify areas within their current role where they can contribute more and propose to take on those tasks. This can involve volunteering for special projects, joining cross-functional teams, or assisting colleagues, who may benefit from their knowledge and experience. By doing so, employees can proactively create opportunities to apply their skills and engage in work that is more meaningful and stimulating [45].

Overqualified employees can seek opportunities for collaboration, mentorship, or coaching that can provide a sense of purpose and engagement in their work. By nurturing social connections, individuals can counteract the negative feelings stemming from perceived overqualification [46]. Employees can reframe their job perceptions and find meaning in their current role. By focusing on the positive aspects of their work, such as opportunities for growth, development, or the contribution they make to the organization, individuals can create a more fulfilling narrative that mitigates the effects of overqualification on boredom [39].

Moreover, modifying the physical or environmental aspects of a job can alleviate job boredom. This can include changes such as rearranging the workspace, adjusting schedules, or seeking new organizational projects or assignments. By altering the context of their work, individuals can introduce novelty and variety, reducing their sense of monotony and boredom [47]. By engaging in job crafting, employees actively shape their work experiences to align themselves better with their skills, interests, and aspirations. This proactive behavior increases job satisfaction and engagement, counteracting the negative impact of perceived overqualification on job boredom [48].

Hypothesis 4 Job crafting behaviors weaken the positive link between POQ and job boredom

### 2.5 Integrated model

In the previous discussion, we established that POQ can lead to job boredom, which mediates the link between POQ and knowledge-hiding. However, we proposed that employees who actively engage in job crafting are less affected by job boredom. Job crafting empowers individuals to proactively shape their work experiences, allowing them to align their tasks and responsibilities with their skills and interests. By actively seeking new challenges and modifying existing tasks, these individuals are less likely to experience the monotony and boredom associated with overqualification. Thus, we hypothesized that job crafting weakens the positive effect between POQ and job boredom, particularly for individuals who actively shape their job roles. The underlying factor that drives the relationship between POQ and knowledge hiding is job boredom. However, this link is expected to be less significant for employees engaged in job crafting. Consequently, the indirect link between perceived overqualification and knowledge hiding is less influenced by job boredom among employees who practice job crafting.

Hypothesis 5 The indirect impact of POQ on knowledge hiding caused by job boredom is moderated by job crafting. As a result, the strength of the indirect effect is minimized when job crafting is high (vs. lower).

## 3 Method

### 3.1 Sample and procedure

The study population comprised employees of 22 hospitality and tourism organizations in Pakistan's northern region. Pakistan's northern region is renowned for its breath-taking natural landscapes, including towering mountain ranges, lush valleys, and pristine lakes. These natural attractions serve as magnets for domestic and international tourists, making the region a hotspot for hospitality and tourism. The data were collected between December 2023–April 2024. The author gained access to study participants by utilizing their professional and personal connections. One of the authors reached out to the human resources departments of each organization to explain the purpose of the research. After receiving official approval, we sent a questionnaire survey to the employees participating in the research. Prior to distributing the questionnaires, they were coded to correspond with employees' responses (T1, T2, and T3). Data collection was completed in three phases, with each phase separated by six weeks. This minimizes the issue of the common method bias [49]. According to Ref. [49], the gap between the data collection phases should not be too short or lengthy. When data collection is too short, the relationship between variables might be exaggerated, but when it is too long, it may lose its significance [50].

We approached 456 employees to fill out questionnaires regarding POQ, job crafting, and demographic variables. In the first phase (T1), 447 responses (98.02 %) were received. In the second wave (T2), after a six-week gap, we approached the same respondents to collect data on job boredom. Four hundred thirty-six responses were returned to their completed surveys (95.61 %). To collect data in the third wave (T3), respondents were approached to collect responses regarding knowledge hiding. In the third wave, 397 questionnaires were received; however, due to missing values, 23 questionnaires were excluded. Finally, 374 (82.01 %) employees' data were used for analysis and interpretation. According to the demographic study, participants had an average age of 34.60 years old, and 67.0 % were male. The majority of the sample (71.2 %) held a Master's degree or higher.

### 3.2 Measures

#### 3.2.1 Perceived overqualification

A nine-item scale developed by Maynard, Joseph [51] was used to measure overqualification. The sample item is “I have a lot of knowledge that I do not need to do my job”.

#### 3.2.2 Knowledge hiding

A twelve-items scale developed by Connelly, Zweig [52] was used to measure knowledge hiding. A sample item is “I offered other members of my team some other information instead of what they wanted.”

#### 3.2.3 Job boredom

The job boredom level was measured using a six-item Dutch boredom scale developed by Reijseger, Schaufeli [53]. Sample items is “I have felt bored at my job".

#### 3.2.4 Job crafting

Job crafting was assessed by using a 15-item scale developed by Tims, Bakker [54]. This scale consists of three dimensions, each dimension further divided into five items. 1) Increasing structural resources. A sample item is “I tried to learn new things at work.”2) Increasing social resources. The sample item is “I asked colleagues for advice.” 3) Increasing challenge job demands. A sample item is “I tried to start new projects at work when there was not much to do.”

#### 3.2.5 Control variables

According to Ref. [55], employee age, gender, educational level and organization tenure affect POQ and knowledge hiding relation. We accounted for the demographics of the employees in our study by controlling for variables such as gender, age, education level, and organizational tenure. Gender was represented as “0 was assigned to male participants” and “1 was assigned to female participants”. The educational level of respondents was evaluated using four distinct parameters, where the values of one, two, three, and four were used to represent HSSC, Bachelor's, Master's, and MS/Phil and PhD degrees, respectively.

## 4 Results

### 4.1 Common method bias

We employed Harman's single-factor test to evaluate the potential for common method bias. This method is used to identify any potential bias that may arise when participants simultaneously respond to predictor and criteria variables [56,57]. In this statistical analysis, we employed an approach that assessed the significance of each variable as a contributing factor. To pass the test, the cumulative variance explained by a single unrotated factor should be below 50 percent to pass the test. Our findings revealed that the total variance explained by a single unrotated factor for the four variables under examination was 29.26 percent, which fell below the 50 percent threshold. These results indicate that the common method bias is not a significant concern in the collected data. Second, we checked for common method bias using a common latent factor (AMOS) as recommended by Podsakoff, MacKenzie [56]. All variables had zero variance similarity. This research did not have an issue with common-method bias. Third, we examined the inner variance inflation factor (VIF) values, as endorsed by Ref. [58], and found VIF values ranging from 1.035 to 1.656, much below the suggested threshold of 3.30 [59]. Our findings show that the common method bias does not substantially impact our findings.

### 4.2 Confirmatory factor analyses

To assess the distinctiveness of the study's four primary variables (POQ, knowledge hiding, job boredom, and job crafting), a series of confirmatory factor analyses (CFAs) were performed. Table 1 displays the results of the CFAs, indicating that the four-factor model demonstrated the best fit among all the models tested: χ2 (69) = 316.34***, RMSEA = 0.04, TLI = 0.90, CFI = 0.91, GFI = 0.93, within the normal range from 0.68 to 0.87 (see Table 2). The average variance extracted (AVE) of the variables range from 0.57 to 0.62, confirming convergent validity [60]. Discriminant validity was checked by comparing the square roots of the average variance extracted with the correlation of the variables. The results show that the AVE values of the variables were greater than the correlations, thus confirming discriminant validity [60]. AVE values were compared with the maximum shared variance (MSV) and average shared variance (ASV) values. According to Ref. [61], discriminant validity is present when all ASV and MSV values are lower than their corresponding AVE values (see Table 2).

**Table 1:** Model's measurement.

Model's	RMSEA	GFI	CFI	TLI	X^2^(df)	ΔX^2^ (df)
(POQ, KH, JC, JB) - M-4	0.04	0.93	0.91	0.90	316.34^a^(69)	–
(POQ&KH, JB, and JC) -*M*-3	0.15	0.77	0.79	0.84	431.21^a^(73)	379.24^a^(3)
(POQ&JB, JC, and KH) - M-3	0.28	0.66	0.65	0.73	534.34^a^(88)	457.21^a^(4)
(POQ&JB&KH, and JC) - M-2	0.32	0.37	0.57	0.54	643.25^a^(83)	533.17^a^(5)
(POQ&JB&KH&JC) - M-1	0.37	0.21	0.35	0.43	767.32^a^(79)	763.22^a^(6)

Note.

a Correlation is significant at the 0.001 level (2-tailed).

**Table 2:** Measurement model analysis summary.

Variables	Factor Loading's	CR	AVE	ASV	MSV
Perceived Overqualification	0.73–0.85	0.93	0.62	0.23	0.36
Knowledge Hiding	0.69–0.87	0.95	0.61	0.27	0.34
Job boredom	0.71–0.77	0.89	0.58	0.26	0.31
Job Crafting	0.73–0.84	0.95	0.58	0.22	0.37

### 4.3 Descriptive statistics

The average scores, SD, correlations, and reliability of the variables are shown in Table 3. The research variables showed good internal consistency and their relationships followed the expected paths. POQ is positively linked to job boredom (r = 0.260, p < 0.01) and knowledge hiding (r = 0.374, p < 0.01). Furthermore, the relationship between job boredom and knowledge hiding was significant (r = 0.323, p < 00.01).

**Table 3:** Mean, SD, correlation and reliability.

Variables	Mean	SD	1	2	3	4
1.Perceived Overqualification	4.06	0.32	**(0.79)**			
2.Knowledge Hiding	3.57	0.52	0.374**	**(0.84)**		
3.Job Boredom	3.55	0.76	0.260**	0.323**	**(0.81)**	
4.Job Crafting	2.28	0.39	−0.031*	0.048	−0.059	**(0.86)**

### 4.4 Hypotheses testing

#### 4.4.1 Direct and mediation path analyses

We used the PROCESS model outlined by Hayes [62] to examine our hypotheses. Table 4 presents the results of this analysis. The analysis supported Hypothesis 1, showing that POQ had a significant positive impact on job boredom (β = 0.60, p < 0.001), indicating that when individuals perceive themselves as overqualified for their jobs, they experience higher levels of boredom. Hypothesis 2 was supported, as the analysis shown that POQ had a significant positive effect on knowledge hiding (β = 0.49, p < 0.001), confirming the hypothesis that perceiving oneself as overqualified increases the tendency to hide knowledge. Hypothesis 3, which involved a mediation test, was examined using Hayes' PROCESS Model 4. The results shown that the relationship between POQ and knowledge hiding was mediated by job boredom. The indirect effect was (β = 0.3234, SE = 0.0737, 95 % CI = 0.4709, 0.1864).

**Table 4:** Hypotheses testing.

Hypotheses	*β*	SE	95 % CI [UL; LL]	*p*	Decision
**Direct effect**					
POQ → Job boredom	0.601	0.062	[0.153; 0.052]	0.000	Accepted
POQ→ Knowledge Hiding	0.492	0.057	[0.174; 0.045]	0.000	Accepted
**Mediating effect**					
POQ → Job boredom→Knowledge Hiding	0.323	0.073	[0.470; 0.186]	0.000	Accepted
**Moderating effect**					
POQ * Job Crafting→ Job boredom	0.793	0.199	[0.401; 0.184]	0.000	Accepted

### 4.5 Moderation: job crafting

**Fig. 2:**
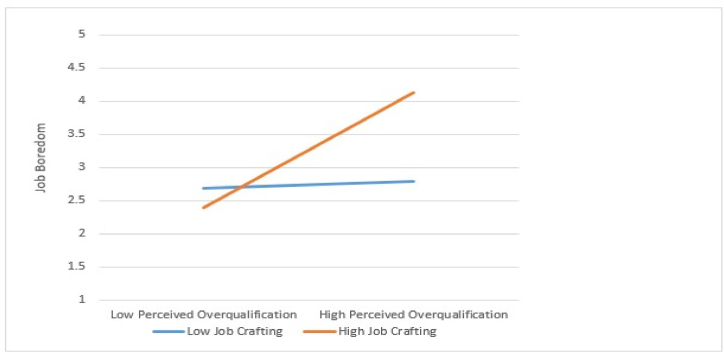
The moderating role of job crafting between POQ and Job boredom.

In addition, Hypothesis 4 investigates the moderating effect of job crafting between POQ and job boredom using Hayes' PROCESS Model 1. According to the findings, job crafting weakened the strength of the positive correlation between the POQ and job boredom (β = 0.7930, SE = 0.1992, p < 00.001, 95 % CI = UL 0.4013; LL 0.1848). These results support Hypothesis 4. Fig. 2 depicts the interaction effect examined to understand the moderating effect of job crafting, and slope tests were utilized to investigate the moderator by analyzing job crafting at +1 and −1 standard deviations from the mean [63]. According to the results, when job crafting is high, the positive relationship between POQ and job boredom is reduced (simple slope = 0.92, SE = 0.1113, p < 00.000) compared to when job crafting is low (simple slope = 0.30, SE = 0.1036, p > 00.005). Based on our findings, we conclude that if employees engage in job crafting activities while performing their duties, the detrimental impact of POQ on job boredom can be mitigated.

### 4.6 Integrated model

To investigate the indirect impact of POQ on knowledge hiding via job boredom, Hayes' PROCESS Model 7 was utilized. The analysis further examined the moderating mediation effect by considering different levels of job crafting (+1 and −1 standard deviation from the mean). When job crafting was high, the indirect effect of POQ on knowledge hiding through job boredom become weakened (indirect effect = 0.5989, SE = 0.1072, 95 % CI = 0.3957, 0.8211), in contrast to situations where job crafting was low (indirect effect = 0.0641, SE = 0.0829, 95 % CI = 0.2286, −0.0966). Consequently, the moderated mediation index demonstrated statistical significance (index = −0.6791, SE = 0.1771, 95 % CI = −1.0363, −0.3398), confirming Hypothesis 5, as reported in Table 5.

**Table 5:** Moderated mediation results.

Moderator	Level	Conditional Indirect Effect	SE	LLCI	ULCI
Job Crafting	Low	0.0641	0.0829	−0.0966	0.2286
	High	0.5989	0.1072	0.3957	0.8211
	Difference	0.5348	0.0243	0.2977	0.5787
Index of moderation mediation		−0.6791	0.1771	−1.0363	−0.3398

**Note**: Moderator values at ± 1 SD.

## 5 Discussion

This study adds a new dimension to our understanding of the complex interplay between POQ, job boredom, and knowledge hiding, underlining the pivotal roles of job boredom and job crafting. Our investigation revealed that overqualified individuals are more prone to experiencing job boredom. Previous research has shown a link between POQ and work-related job boredom [64]. Overqualified employees may experience a lack of engagement and stimulation in their roles, leading to feelings of boredom [65]. Studies have indicated that when individuals believe that their skills and abilities exceed the requirements of their jobs, they are more likely to experience dissatisfaction, leading to higher levels of boredom at work [28]. Moreover, work-related job boredom can also contribute to knowledge hiding by explaining the mediating path between POQ and knowledge hiding [66]. Overqualified employees who experience boredom might engage in knowledge hiding to cope with their negative emotions, or as a form of passive resistance. Boredom can reduce motivation to contribute to the organization, leading to a lack of willingness to share knowledge [67].

The study results show that POQ leads to knowledge hiding, and such results align with those of previous studies [68]. The idea that overqualified individuals may engage in knowledge hiding as a result of their dissatisfaction is supported by research on organizational justice and behavioral responses [69]. POQ can lead to a sense of relative deprivation or perceived unfairness, which can lead to negative behaviors, such as knowledge hiding [11]. Employees who feel undervalued or underutilized may withhold information and expertise to regain a sense of control or protect themselves from exploitation [70].

Incorporating job crafting into this relationship could mitigate these adverse outcomes. Research suggests that when individuals have the autonomy to shape their job roles, they experience higher job satisfaction and engagement [45]. Job crafting can provide control over tasks and reduce feelings of overqualification and job boredom, consequently lowering the likelihood of engaging in knowledge hiding behaviors [71,72].

According to relative deprivation theory, overqualified individuals may experience feelings of resentment or dissatisfaction [73]. Relative deprivation theory aligns with the idea that overqualified individuals might experience negative emotions due to a perceived mismatch between their qualifications and job roles [74]. These negative emotions can cascade into feelings of job boredom and contribute to behaviors such as knowledge hiding [13]. This study investigates this phenomenon to examine whether this sense of overqualification also increase job boredom and, consequently, knowledge hiding behaviors [75]. Our research findings enrich the existing literature on the impact of POQ on workplace behavior. This emphasizes the significant mediating role of job boredom and the moderating role of job crafting in comprehending the complex relationship between POQ and knowledge hiding.

### 5.1 Theoretical implications

First, the literature has investigated the concept of perceived overqualification (POQ) within the context of negative outcomes, such as diminished job satisfaction, decreased task performance, or tendencies toward withdrawal. However, this study offers a distinct perspective by establishing a link between POQ and knowledge hiding in the hospitality and tourism sectors. This unexplored relationship introduces an intricate dynamic between an individual's perception of their overqualification and the resulting unethical workplace behavior. Thus, it enriches our understanding of POQ's potential implications, revealing that it can spur not just feelings of discontent but also behaviors such as knowledge hiding that can significantly impair a collaborative and productive work atmosphere [76].

Second, job boredom is a relatively under-researched topic in organizational behavior compared to job attitudes, such as anger, deprivation, and burnout. However, this study also provides valuable insights into the role of job boredom. This study posits that job boredom mediates the relationship between POQ and knowledge hiding. This is a novel contribution, suggesting that the feeling of underutilization among overqualified employees can lead to job boredom, which in turn prompts detrimental behaviors such as knowledge hiding. This study also highlights the potential for job boredom when employees perceive themselves as overqualified. This extends our understanding of the causes of job boredom beyond mundane or repetitive tasks, and indicates that mismatches between employee qualifications and job requirements can also contribute to job boredom.

Third, job crafting is the proactive behavior of employees to shape and mold their jobs to fit their personal abilities, needs, and preferences. Previous research has largely focused on how job crafting impacts job crafters, with less emphasis on how it might influence broader work contexts. This study highlights the role of job crafting as a moderating variable that can reduce the negative impact of POQ. Specifically, the study suggests that by providing autonomy and flexibility for employees to craft their jobs, they might feel less overqualified and less likely to hide their knowledge. This places job crafting as a potential strategic tool for managing the adverse effects of POQ and emphasizes the role of proactive employee behavior in shaping job outcomes [77].

Fourth, the hospitality and tourism industry often faces unique challenges, including high employee turnover rates, diverse job roles, and a pressing need for knowledge sharing to ensure smooth operation. The finding that a POQ can lead to knowledge hiding in this specific industry underscores the need for effective management practices tailored to these unique industry conditions. Moreover, the moderating role of job crafting in this industry context implies that managers in the hospitality and tourism sectors may need to focus more on providing employees with opportunities to shape their job roles. This industry-specific perspective adds another layer of value to this study, allowing for more nuanced and contextualized insights.

### 5.2 Practical implications

First, the HR department (Human Resources department) should pay particular attention to the recruiting process used to choose candidates so that they select those who best match the position rather than those who are overqualified. Human resources departments should present detailed job previews to candidates throughout the recruiting process so that they can obtain a thorough picture of the position. Second, to avoid POQ, managers must make every effort to allocate human resources optimally and put subordinates in the most effective roles. Third, supervisors must watch shifts in their employees' mindsets and modify their roles as soon as such changes are shown. Fourth, job crafting is particularly important for highly qualified individuals. "Job crafting” refers to proactively and independently altering one's working conditions to suit one's needs, goals, and abilities and reducing knowledge hiding. Fifth, job crafting as a moderator provides important options for career and professional development. To promote employee well-being and performance, organizations must empower workers with opportunities for self-governance in the workplace. Highly skilled individuals benefit from a more diverse work environment because they reduce boredom and boost morale and productivity. Interventions to improve the person-work fit and foster a sense of purpose are known as “job crafting.” Many job crafting treatments match employment to workers' interests, skills, and abilities [26,78].

Recently, Hu, McCune Stein [79] presented a novel paradigm of job crafting known as “job constructing toward skills and interest.” This technique helps to better match workers' resources and job requirements. The early results of a job crafting intervention based on this approach showed that it enhanced person–job fit, particularly for employees in their later years. This was especially true for workers who had been in the workforce for a longer period. Employees' potential and sense of purpose may be maximized using these tactics in conjunction with top-down activities (e.g., recruiting, selection, training, and development). Organizations must engage in human capital selection and development methods to bring in the finest candidates for open positions, select and keep them, and help them grow and improve their skills.

### 5.3 Limitation and future direction

First, self-reported ratings are often used in research, and drawbacks such as bias and exaggerated replies must be fixed. Colleagues, supervisors, and data from archived sources should be included in future studies to reduce systemic bias. Second, conducting similar studies in different industries or cultures can help to test the generalizability of our findings. This will allow you to see the results across different contexts. Further research could apply the results of this study to other industries. Third, we examined employees of all ages in Pakistani culture to draw conclusions. According to Ref. [80], boredom is a subjective feeling impacted by various demographic parameters, such as gender and age.

Furthermore, a considerable proportion of young employees think that they are overqualified for their jobs [81]. Consider including a diverse sample of participants from different age groups and sociocultural backgrounds. This will help to ensure that the findings are not limited to a specific population subset. The younger generation has a stronger sense of being overqualified. As a means to a better-paying career, many young people are obligated to take on positions in which they are overqualified. Fourth, knowledge hiding behavior is difficult to evaluate objectively and effectively because of its implicit nature [82]. Self-reported knowledge hiding questionnaires may have led employees to underestimate the knowledge they hide in this study. In the future, researchers should conduct interviews to analyze the behavior of workers who hide knowledge to gain more reliable data about employee knowledge hiding.

## 6 Conclusion

The results show a positive link between POQ and knowledge hiding, where employees intentionally hide knowledge because of person-job misfit. Job boredom mediates the relationship between POQ and knowledge hiding. This suggests that when employees feel overqualified for their roles, they may experience boredom because they are not sufficiently challenged or engaged. This boredom, in turn, could lead them to hide their knowledge rather than actively contribute to their work environments. The study also found that job crafting moderated the link between POQ and job boredom. Thus, employees engaging in job crafting are less likely to experience boredom. Overall, this study contributes to our understanding of the factors contributing to knowledge hiding in the workplace and highlights the importance of considering employees' perceptions of their qualifications and job crafting behaviors when designing interventions to reduce knowledge hiding.

## Data availability statement

The data will be available on request form the corresponding author.

## Funding information

This research was supported by Key Project of National Social Science Foundation of China (21AGL014); Shenzhen Science and Technology Program (JCYJ20210324093208022); 10.13039/501100009019Shenzhen University Humanities and Social Sciences High-level Innovation Team Project for Leading Scholars (24LJXZ06).

## CRediT authorship contribution statement

**Jawad Khan:** Writing – original draft, Formal analysis, Data curation, Conceptualization. **Qingyu Zhang:** Writing – review & editing, Writing – original draft, Supervision, Funding acquisition, Formal analysis, Data curation, Conceptualization. **Imran Saeed:** Writing – original draft, Investigation, Funding acquisition. **Amna Ali:** Visualization, Validation. **Mohammad Fayaz:** Writing – review & editing, Methodology.

## Declaration of competing interest

The authors declare that they have no known competing financial interests or personal relationships that could have appeared to influence the work reported in this paper.
