# Supplementary material for: Unveiling the nexus between perceived overqualification and knowledge hiding: Moderated mediation analysis of job crafting and job boredom
Source: Heliyon. 2024 May 22;10(10):e31701. doi: 10.1016/j.heliyon.2024.e31701 (PMC11145549; doi:10.1016/j.heliyon.2024.e31701)
Supplement: Multimedia component 1 [file mmc1.docx]

**Questionnaire**

**Age:** ______________ **Gender:** M / F

**Marital Status:** Single / Married **Qualification:** Doctorate/ Masters/ Bachelors

**Tenure with current organization:** __________

**Perceived Overqualification**

1. My job requires less education than I have

| Strongly Disagree | Disagree | Neutral | Agree | Strongly Agree |
| --- | --- | --- | --- | --- |

1. The work experience that I have is not necessary to be successful on this job

| Strongly Disagree | Disagree | Neutral | Agree | Strongly Agree |
| --- | --- | --- | --- | --- |

1. I have job skills that are not required for this job

| Strongly Disagree | Disagree | Neutral | Agree | Strongly Agree |
| --- | --- | --- | --- | --- |

1. Someone with less education than myself could perform well on my job

| Strongly Disagree | Disagree | Neutral | Agree | Strongly Agree |
| --- | --- | --- | --- | --- |

1. My previous training is not being fully utilized on this job

| Strongly Disagree | Disagree | Neutral | Agree | Strongly Agree |
| --- | --- | --- | --- | --- |

1. I have a lot of knowledge that I do not need in order to do my job

| Strongly Disagree | Disagree | Neutral | Agree | Strongly Agree |
| --- | --- | --- | --- | --- |

1. My education level is above the education level required by my job

| Strongly Disagree | Disagree | Neutral | Agree | Strongly Agree |
| --- | --- | --- | --- | --- |

1. Someone with less work experience than myself could do my job just as well

| Strongly Disagree | Disagree | Neutral | Agree | Strongly Agree |
| --- | --- | --- | --- | --- |

1. I have more abilities than I need in order to do my job

| Strongly Disagree | Disagree | Neutral | Agree | Strongly Agree |
| --- | --- | --- | --- | --- |

**Knowledge Hiding**

1. I agreed to help him/her but never really intended to

| Strongly Disagree | Disagree | Neutral | Agree | Strongly Agree |
| --- | --- | --- | --- | --- |

1. I agreed to help him/her but instead gave him/her information different from what s/he wanted

| Strongly Disagree | Disagree | Neutral | Agree | Strongly Agree |
| --- | --- | --- | --- | --- |

1. I told him/her that I would help him/her out later but stalled as much as possible

| Strongly Disagree | Disagree | Neutral | Agree | Strongly Agree |
| --- | --- | --- | --- | --- |

1. I offered him/her some other information instead of what he/she really wanted

| Strongly Disagree | Disagree | Neutral | Agree | Strongly Agree |
| --- | --- | --- | --- | --- |

1. I pretended that I did not know the information

| Strongly Disagree | Disagree | Neutral | Agree | Strongly Agree |
| --- | --- | --- | --- | --- |

1. I said that I did not know, even though I did to

| Strongly Disagree | Disagree | Neutral | Agree | Strongly Agree |
| --- | --- | --- | --- | --- |

1. I pretended I did not know what s/he was talking about

| Strongly Disagree | Disagree | Neutral | Agree | Strongly Agree |
| --- | --- | --- | --- | --- |

1. I said that I was not very knowledgeable about the topic

| Strongly Disagree | Disagree | Neutral | Agree | Strongly Agree |
| --- | --- | --- | --- | --- |

1. I explained that I would like to tell him/her, but was not supposed to

| Strongly Disagree | Disagree | Neutral | Agree | Strongly Agree |
| --- | --- | --- | --- | --- |

1. I explained that the information is confidential and only available to people on a particular project

| Strongly Disagree | Disagree | Neutral | Agree | Strongly Agree |
| --- | --- | --- | --- | --- |

1. I told him/her that my boss would not let anyone share this knowledge

| Strongly Disagree | Disagree | Neutral | Agree | Strongly Agree |
| --- | --- | --- | --- | --- |

1. I said that I would not answer his/her questions

| Strongly Disagree | Disagree | Neutral | Agree | Strongly Agree |
| --- | --- | --- | --- | --- |

**Job Crafting**

1. I try to develop my capabilities

| Strongly Disagree | Disagree | Neutral | Agree | Strongly Agree |
| --- | --- | --- | --- | --- |

1. I try to develop myself professionally

| Strongly Disagree | Disagree | Neutral | Agree | Strongly Agree |
| --- | --- | --- | --- | --- |

1. I try to learn new things at work

| Strongly Disagree | Disagree | Neutral | Agree | Strongly Agree |
| --- | --- | --- | --- | --- |

1. I make sure that I use my capacities to the fullest

| Strongly Disagree | Disagree | Neutral | Agree | Strongly Agree |
| --- | --- | --- | --- | --- |

1. I decide on my own how I do things

| Strongly Disagree | Disagree | Neutral | Agree | Strongly Agree |
| --- | --- | --- | --- | --- |

1. I ask my supervisor to coach me

| Strongly Disagree | Disagree | Neutral | Agree | Strongly Agree |
| --- | --- | --- | --- | --- |

1. I ask whether my supervisor is satisfied with my work

| Strongly Disagree | Disagree | Neutral | Agree | Strongly Agree |
| --- | --- | --- | --- | --- |

1. I look to my supervisor for inspiration

| Strongly Disagree | Disagree | Neutral | Agree | Strongly Agree |
| --- | --- | --- | --- | --- |

1. I ask others for feedback on my job performance

| Strongly Disagree | Disagree | Neutral | Agree | Strongly Agree |
| --- | --- | --- | --- | --- |

1. I ask colleagues for advice

| Strongly Disagree | Disagree | Neutral | Agree | Strongly Agree |
| --- | --- | --- | --- | --- |

1. When an interesting project comes along, I offer myself proactively as project co-worker

| Strongly Disagree | Disagree | Neutral | Agree | Strongly Agree |
| --- | --- | --- | --- | --- |

1. If there are new developments, I am one of the first to learn about them and try them out

| Strongly Disagree | Disagree | Neutral | Agree | Strongly Agree |
| --- | --- | --- | --- | --- |

1. When there is not much to do at work, I see it as a chance to start new projects

| Strongly Disagree | Disagree | Neutral | Agree | Strongly Agree |
| --- | --- | --- | --- | --- |

1. I regularly take on extra tasks even though I do not receive extra salary for them

| Strongly Disagree | Disagree | Neutral | Agree | Strongly Agree |
| --- | --- | --- | --- | --- |

1. I try to make my work more challenging by examining the underlying relationships between aspects of my job

| Strongly Disagree | Disagree | Neutral | Agree | Strongly Agree |
| --- | --- | --- | --- | --- |

**Job Boredom**

1. At work, time goes by very slowly a

| Strongly Disagree | Disagree | Neutral | Agree | Strongly Agree |
| --- | --- | --- | --- | --- |

1. At my work, there is not so much to do

| Strongly Disagree | Disagree | Neutral | Agree | Strongly Agree |
| --- | --- | --- | --- | --- |

1. I feel bored at my job a

| Strongly Disagree | Disagree | Neutral | Agree | Strongly Agree |
| --- | --- | --- | --- | --- |

1. Are there long periods of boredom on the job?

| Strongly Disagree | Disagree | Neutral | Agree | Strongly Agree |
| --- | --- | --- | --- | --- |

1. Does the time seem to go by slowly?

| Strongly Disagree | Disagree | Neutral | Agree | Strongly Agree |
| --- | --- | --- | --- | --- |

1. At work, I spend my time aimlessly

| Strongly Disagree | Disagree | Neutral | Agree | Strongly Agree |
| --- | --- | --- | --- | --- |
